# Molecular analysis of fungal populations in patients with oral candidiasis using next-generation sequencing

**DOI:** 10.1038/srep28110

**Published:** 2016-06-16

**Authors:** Yumi Imabayashi, Masafumi Moriyama, Toru Takeshita, Shinsuke Ieda, Jun-Nosuke Hayashida, Akihiko Tanaka, Takashi Maehara, Sachiko Furukawa, Miho Ohta, Keigo Kubota, Masaki Yamauchi, Noriko Ishiguro, Yoshihisa Yamashita, Seiji Nakamura

**Affiliations:** 1 Section of Oral and Maxillofacial Oncology, Division of Maxillofacial Diagnostic and Surgical Sciences, Faculty of Dental Science, Kyushu University, Fukuoka, Japan; 2 OBT Research center, Faculty of Dental Science, Kyushu University, Fukuoka, Japan; 3 Section of Preventive and Public Health Dentistry, Division of Oral Health, Growth and Development, Faculty of Dental Science, Kyushu University, Fukuoka, Japan; 4 Department of Oral and Maxillofacial Surgery, Wakayama Medical University, School of Medicine, Wakayama, Japan

## Abstract

Oral candidiasis is closely associated with changes in oral fungal biodiversity and is caused primarily by *Candida albicans*. However, the widespread use of empiric and prophylactic antifungal drugs has caused a shift in fungal biodiversity towards other *Candida* or yeast species. Recently, next-generation sequencing (NGS) has provided an improvement over conventional culture techniques, allowing rapid comprehensive analysis of oral fungal biodiversity. In this study, we used NGS to examine the oral fungal biodiversity of 27 patients with pseudomembranous oral candidiasis (POC) and 66 healthy controls. The total number of fungal species in patients with POC and healthy controls was 67 and 86, respectively. The copy number of total PCR products and the proportion of non-*C. albicans,* especially *C. dubliniensis*, in patients with POC, were higher than those in healthy controls. The detection patterns in patients with POC were similar to those in controls after antifungal treatment. Interestingly, the number of fungal species and the copy number of total PCR products in healthy controls increased with aging. These results suggest that high fungal biodiversity and aging might be involved in the pathogenesis of oral candidiasis. We therefore conclude that NGS is a useful technique for investigating oral candida infections.

Oral candidiasis is considered to be caused primarily by *Candida* (*C.*) *albicans* and is closely associated with changes in fungal biodiversity. However, the widespread use of empiric and prophylactic antifungal drugs in recent years has caused a change in fungal biodiversity among other yeast species, including non- *Candida albicans* species (e.g., *C. glabrata*, *C. tropicalis*, *C. krusei*) and non-*Candida* species (e.g. *Trichosporon sp, Malassezia sp, Cladosporium sp, Aspergillus sp*)^1^. A change in fungal biodiversity is also associated with long-term treatment with antifungal drugs^2^. In view of the drug resistance profiles of fungal pathogens, the development of rapid methods for species-specific identification is desirable to permit selection of the most appropriate antifungal treatment^3^. Although phenotypic analysis of fungal cultures is the traditional approach to the identification of fungal species, this approach is time consuming and shows limited applicability for the detection of molds^4^. Recent advances in high-throughput DNA sequencing, or next-generation sequencing (NGS), has allowed the knowledge of bacterial biodiversity to be refined and redefined. NGS provides a large number of sequence reads in a single rapid run and detects not only the most dominant community but also the low-abundance or rare taxa^5^,^6^.

Over the last decades, many studies of oral microbial diversity have been undertaken, using 16S rRNA-based analysis by NGS^7^. However, to our knowledge, no published reports have investigated the oral fungal biodiversity using 18S rRNA-based analysis by NGS. In this study, we have established a new method for exhaustive analysis of the oral fungal population using NGS. Using this method, we then examined the oral fungal biodiversity of patients with oral candidiasis and also healthy subjects, providing a detailed characterization of oral fungal biodiversity in health and disease.

## Materials and Methods

### Ethics Statement

The study design and methods were approved by the Institutional Review Board of Center for Clinical and Translational Research of Kyushu University Hospital (IRB number: 25–269). The methods were carried out in accordance with the approved guidelines. All patients or their parents gave their written informed consent within written treatment contract on admission and therefore prior to their inclusion in the study.

### Study Participants

The participants of this study consisted of 66 healthy individuals (26 men and 40 women; mean age, 38.7 ± 12.6 years) and 27 patients with pseudomembranous oral candidiasis (POC) (3 men and 24 women; mean age, 64.2 ± 14.5 years) who were referred to the Department of Oral and Maxillofacial Surgery, Kyushu University Hospital, Japan, between 2012 and 2015.

The healthy individuals were selected with the following inclusion criteria (a) non-smokers, (b) non-denture wearers, and (c) no clinical signs of oral mucosal disease such as xerostomia or reduced saliva production. Exclusion criteria were (a) systemic diseases, (b) receiving antibiotic or steroid therapy in the last 6 months, (c) pregnancy or breastfeeding, and (d) having any allergies. After diagnosis by direct microscopy, the patients started therapy. Florid oral gel (FLO-G) 2% 2.5–5 g (miconazole, Mochida Pharmaceutical Co., Ltd. Tokyo, Japan) was applied to the oral mucosa four times a day (after meals and before sleep). The treatment period was defined as follows: antifungal therapy was continued until white patches on the oral mucosa were resolved and subjective complaints, including burning pain or haphalgesia, ceased. The average treatment period for POC was 24.8 ± 10.8 days. Fifteen of the 27 patients were available for follow-up after complete recovery.

### Sampling of oral rinse

Sampling of oral rinse was performed according to a protocol described previously^8^. Briefly, oral rinse samples were collected at least 1 h after a meal and also after tooth brushing. Study participants rinsed their mouth with 5 mL of saline for 30 s and expectorated into a sterile plastic 50-mL tube. The collected samples were centrifuged at 9600 g for 10 min at 4 °C to harvest the cell pellets. The cell pellets were stored at −80 °C until processing.

### DNA Extraction

DNA extraction from oral rinse was performed according to a protocol described previously^9^. Cell pellets were re-suspended in 5 mL phosphate-buffered saline. Three milliliters of each sample was centrifuged at 9600 g for 10 min at 4 °C. The pellets were re-suspended in 800 μL of 0.2% sodium dodecyl sulfate (SDS) and 200 μL of 10 mM Tris-HCl/1 mM EDTA/pH 8.0 (TE), and incubated with 20 μg RNase (Sigma-Aldrich Co. LLC) at 15 °C for 10 min. The samples were then filtered through a 79-mm filter (Nippon-Clever Co. Ltd., Aichi, Japan) fixed in a 13-mm Swinny stainless filter holder (Merck Millipore, Darmstadt, Germany). The filtered samples were centrifuged at 2330 g for 10 min at 4 °C. The pellets were re-suspended in 400 mL of 1% SDS in TE and incubated with 10 μg of RNase at 37 °C for 10 min. Zirconia-silica beads (0.3 g, bead size 0.1 mm; Biospec Products, Bartlesville, OK, USA) and one tungsten carbide bead (bead size 3 mm; Qiagen) were added to each sample. The samples were incubated at 55 °C for 10 min, followed by violent agitation for 3 min in a cell disruptor (Disruptor Genie; Scientific Industries, Inc., Bohemia, NY, USA). Then, 150 μL 1% SDS was added to each sample and incubated at 70 °C for 10 min. After adding 300 μL phenol and 600 μL chloroform, the supernatant liquid was then collected by centrifugation at 10600 g for 10 min at 4 °C and a further 600 μL chloroform was added. DNA was precipitated by addition of 0.1× volumes of 3 M NaAc and 1× volume of 2-propanol, incubation at −20 °C overnight, and centrifugation at 21600 g for 15 min. Following centrifugation, the DNA was rinsed with 70% ethanol and re-suspended in 30 μL TE. DNA samples were kept at −20 °C until analysis.

### Cultivation

One milliliter of the re-suspended cell pellets from the oral rinse was immediately centrifuged at 9600 g for 5 min at 4 °C. The pellets were re-suspended in 250 μL of TE, and 100 μL of the sample was spread onto CHROMagar plates containing 0.5 mg chloramphenicol per mL (BBL CHROMagar Candida, Becton, Dickinson and Company, Franklin Lakes, NJ, USA). Plates were incubated at 37 °C for 48 h. *Candida* species were determined by colony color.

### Quantitative PCR Analysis

We quantified the fungal populations using real-time PCR of the fungal internal transcribed spacer (ITS) 1 region in the final DNA samples. Quantitative real-time PCR was performed with the SYBR Green PCR kit (Qiagen, Hilden, Germany) in the 7500 Real-Time PCR System (Applied Biosystems, Foster City, CA, USA) following the manufacturer’s instructions. The primers we used were ITS1-F (5′-CTT GGT CAT TTA GAG GAA GTA A-3′) and ITS2 (5′-CGC TGC GTT CTT CAT CG-3′). The cycling conditions were 95 °C for 10 min, followed by 60 cycles of 95 °C for 3 s, and annealing and extension at 65 °C for 30 s. The ITS1 region of *C. albicans* was inserted into the vector plasmid pBluescript SK II (+) (Stratagene, La Jolla, CA, USA) and used as a real-time control.

### Fusion PCR

Amplification of the ITS1 region was performed using the forward primers ITS1F with sequencing adaptor and the reverse primers ITS2. PCR reactions comprised 1 μL of DNA, 1 U of KOD Plus Ver.2 polymerase (Toyobo, Osaka, Japan), and 10 pmol of each primer making a total reaction volume of 10 μL. Amplification was performed in the following conditions - 94 °C for 3 min, followed by 35 cycles of 94 °C for 15 s, 60 °C for 30 s, and 68 °C for 60 s. PCR products were purified using the Agecourt AMpure XP Kit. The purified products were separated by electrophoresis on a 1.3% agarose gel, bands were cut out in a region between 200 and 400 bp, and subsequently purified using the Qiagen Gel Purification Kit (Qiagen, Hilden, Germany). After purification, 1 μL of purified PCR product was analyzed by capillary electrophoresis on a chip to confirm the expected fragment size was obtained. DNA concentration was determined using the KAPA SYBR Fast qPCR Kit (KAPA Biosystems, Wilmington, MA, USA). All amplicons were diluted to 13 pM in 30 μL TE prior to library preparation.

### Library Preparation and Sequencing

Emulsion PCR was performed on the One Touch Instrument (Life Technologies, Carlsbad, CA, USA) using the Ion PGM Template OT2 400 Kit (Life Technologies) according to the manufacturer’s protocol. Sequencing was performed on the Personal Genome Machine (PGM) using the Ion PGM Sequencing 400 Kit with a 314 V2 chip according to the manufacturer’s protocol.

### Data Analysis and Taxonomy Assignment

The sequenced Ion Torrent raw FASTQ reads were imported into CLC Genomics Workbench v.7.5.2 (CLC bio; Aarhus, Denmark), and were first trimmed to the quality score limit 0.01 with no ambiguous nucleotides. Sequences obtained from all samples were analyzed together. Sequences were excluded from the analysis using a script written in R if they were shorter than 200 bases or if they did not include the correct forward primer sequence. For each sequence, nearest-neighbor species with ≥98% identity were selected as candidates using BLAST searches against 22774 oral fungi ITS region sequences in the UNITE (Unified system for the DNA based fungal species (ver. 7.0) database.

### Statistical Analysis

The significance of differences between groups was determined using χ^2^ tests and Student t tests. A P-value <0.05 was considered statistically significant. All statistical analyses were performed using JMP software (V.8; SAS Institute, Cary, NC, USA).

## Results

### Quantification of Fungal Flora in Patients with POC and Controls

Real-time PCR analysis and cultivation on CHROMagar Candida plates were performed to quantify the fungal flora in oral rinse samples from 27 POC patients and 66 healthy controls. As shown in Table 1, the total number of PCR products from DNA samples and the number of samples exhibiting colonization by *Candida* species were significantly higher in patients with POC than those in controls. Furthermore, the total number of PCR products and the number of samples colonized by *Candida* species in the control group increased with aging.

The detection rate of *Candida* species identified by this cultural method is as follows: *C. albicans* (POC, 100%; controls, 100%), *C. parapsilosis* (POC, 9.7%; controls, 0%)*, C. tropicalis* (POC, 12.9%; controls, 0%)*, C. krusei* (POC, 9.7%; controls, 0%), and *C. glabrata* (POC, 11.8%; controls, 7.6%).

### Species Diversity of Fungal Populations in Patients with POC and Controls

**Figure 1:**
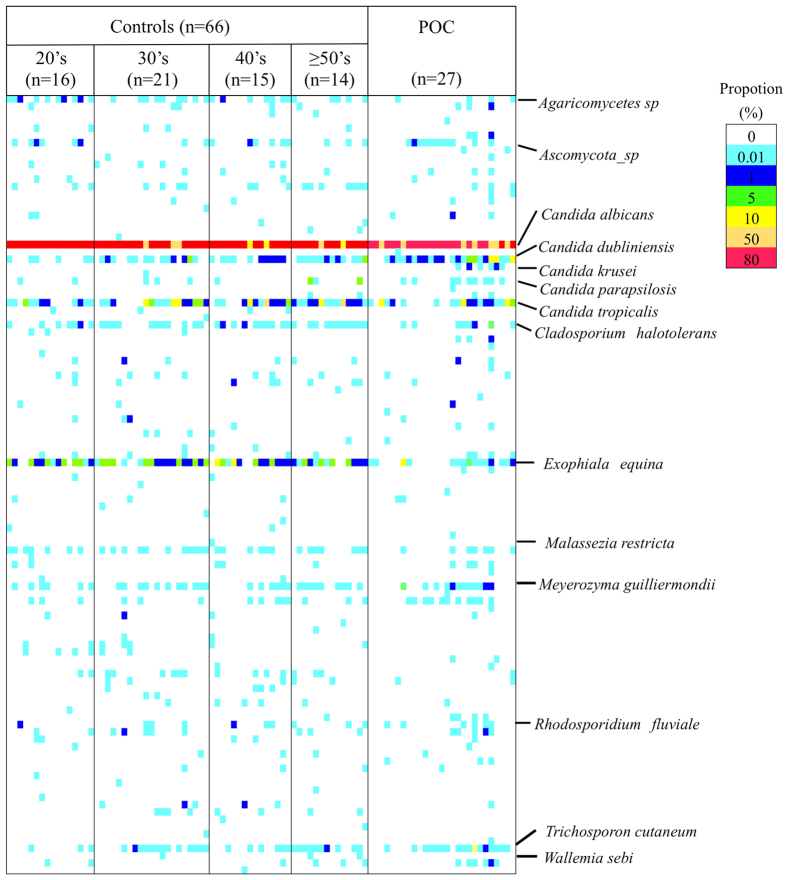
Fungal population in patients with pseudomembranous oral candidiasis (POC) and controls visualized as a gel-like image by using next-generation sequencing (NGS). The proportion (107 detected species) in the NGS profile is represented by the color-scale intensity of each grid.

NGS analysis of the ITS1 region was conducted to assess the diversity of fungal species in patients with POC and controls. The total and average numbers of fungal species per person were higher in controls than those in patients with POC (Table 1). The NGS profiles are displayed as a gel-like image in Fig. 1. One hundred and seven species were identified by NGS. The details of all 108 species are as follows: 45 species were common to both the patients with POC and the controls; 22 species were identified only in the patients with POC; 41 species were identified only in the controls. Table 2 showed the detection ratios of fungal populations from patients with POC and controls. *C. albicans* was identified in all participants. In contrast, *C. dubliniensis*, *C. parapsilosis*, *Wallumia sebi*, *Rhodosporidium babjevae*, *C. krusei*, *Antrodiella micra*, *Cladosporium sphaerospermum*, and *Sporidiobolales* species were detected at a significantly higher rate in POC patients than in controls, whereas *Exophiala equina*, *Cladosporium halotolerans*, and *Agaricomycetes* species were detected at a significantly higher rate in controls.

**Figure 2:**
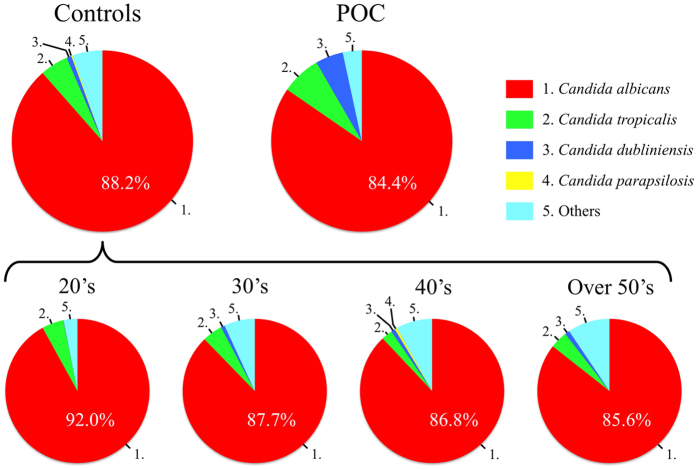
The mean composition ratio of fungal populations from controls and patients with POC. Top 4 fungi of detection rate in control group are shown.

With regard to the composition ratio of fungal populations, *C. albicans* constituted more than 80% of the total fungal populations in both groups. Interestingly, the proportion of *C. dubliniensis* in patients with POC was higher than those in controls (POC, 4.9%; controls, 0.9%). Moreover, the proportion of non-*C. albicans* in the controls gradually increased with age (Fig. 2).

### Change in Fungal Populations Following Antifungal Therapy

**Figure 3:**
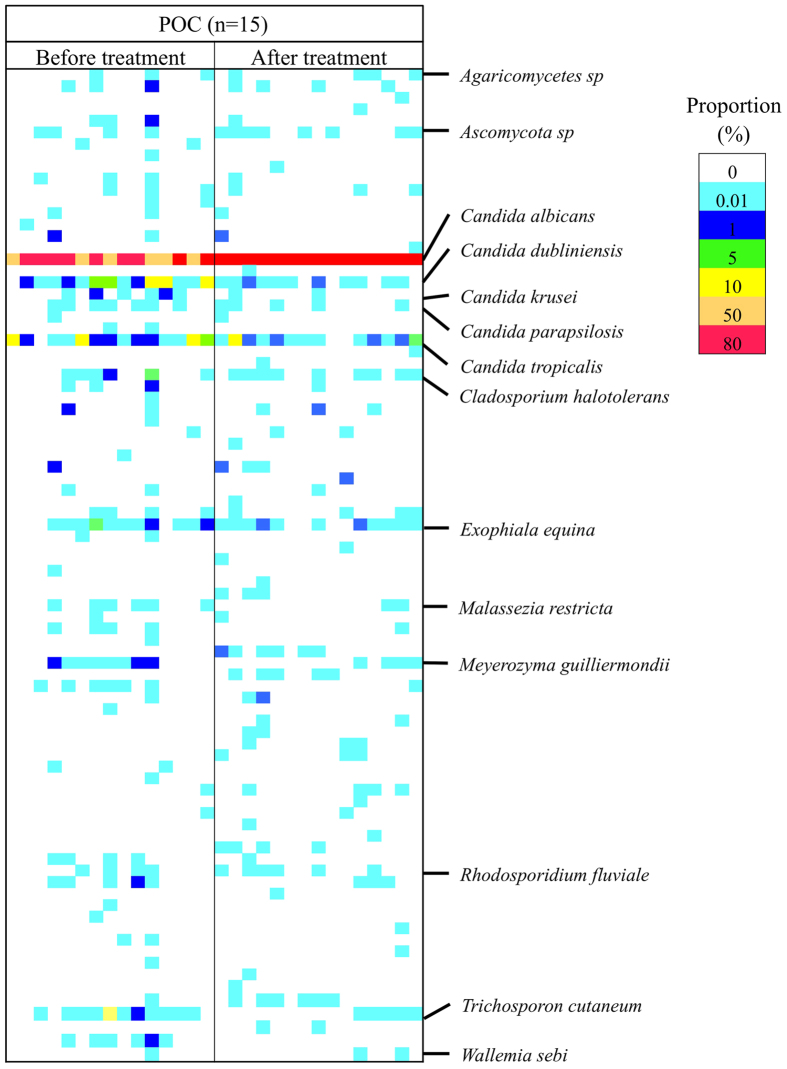
Fungal population in patients with POC before and after therapy visualized as a gel-like image by NGS. The proportion (65 detected species) in the NGS profile is represented by the color-scale intensity of each grid.

**Figure 4:**
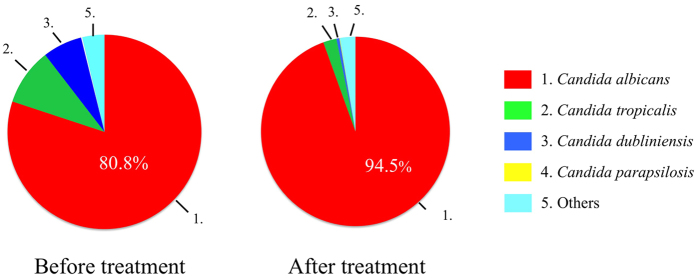
The mean composition ratio of fungal populations from patients with POC before and after therapy. Top four fungi of the detection rate in POC before the treatment group are shown.

Fifteen of 27 patients with POC were available for serial assessments following antifungal treatment with FLO-G. The NGS profiles are displayed as a gel-like image in Fig. 3. After treatment, the detection patterns in patients with POC became similar to those in controls. Although the copy number of total PCR products by real time PCR significantly increased, the total and average numbers of fungal species per person increased (Table 1). In addition, the proportion of non-*C. albicans*, especially *C. dubliniensis*, considerably decreased (Fig. 4).

## Discussion

The oral microbiome, which includes oral fungi, is one of the most complex and diverse in the human body. Oral fungi can either prevent or cause infections. With a disturbance of fungal homeostasis, an increase in the pathogenic community can promote opportunistic infections^10^,^11^,^12^,^13^. *Candida* species, especially *C. albicans* is the most commonly identified fungus in the oral cavity and is considered a major pathogen of oral candidiasis^14^,^15^. However, several studies indicated that non-*Candida* species could also be pathogenic, causing altered diversity of the oral fungal flora with an increase in resistant species against antifungal agents^16^,^17^.

Traditionally, the fungal flora has been examined using cultivation-based methods such as CHROMagar Candida, which, based on the color of colonies, can be used to identify five *Candida* species: *C. albicans, C. parapsilosis, C. tropicalis, C. krusei*, and *C. glabrata.* In this study, the detection rate of these *Candida* species by this culture methods were generally similar to that by NGS. However, this method allows identification of the most common *Candida* species but it is difficult to identify other less common fungal species. Our previous studies demonstrated the utility of length heterogeneity-polymerization chain reaction (LH-PCR) analysis of ITS1 regions of fungal nuclear 18s rRNA in oral candidiasis. More than 40 species of oral fungi could be detected in patients with POC by LH-PCR analysis and revealed that the diversity of fungal flora including *C. dubliniensis* and other *Candida* species could be important in the pathogenesis of oral candidiasis. However, using this method, prior knowledge of individual nucleotide sequences of ITS1 fragments is necessary to identify their fungal origins. Recently, NGS methods surveying 16S rRNA in oral microbial flora have been developed and revealed a vast complexity of species^5^,^18^. In this study, we thus evaluated the fungal biodiversity from patients with POC using NGS analysis of the ITS1 region of fungal nuclear 18S rRNA. This NGS analysis enabled the detection of more than 80 species of oral fungi compared with only around 40 by LH-PCR, and without prior knowledge of the nucleotide sequences^17^. Our current NGS data indicate that the increase in numbers and diversity of specific *Candida* species, including *C. dubliniensis*, *C. parapsilosis*, and *C. krusei*, might be involved in the pathogenesis of POC. These results are consistent with our previous studies of LH-PCR.

In contrast, the number of fungal species identified by NGS was significantly lower in patients with POC than in controls, which is opposite to the results obtained by LH-PCR. This discrepancy may be due to one of the following reasons: (1) patients with POC increased some specific fungi and decreased other fungi compared with controls; (2) LH-PCR is limited to the detection of some specific fungi; (3) NGS could detect both some specific fungi and other negligible fungi. Interestingly, the copy number of total PCR products, the number of identified fungal species, and the proportion of non-*C. albicans* in controls increased with age. These results suggest that aging might be associated with oral fungal biodiversity and the initiation of POC.

In this study, NGS analysis of the ITS1 region of fungi was performed because NGS does not have the ability to sequence reads over 400 bp, which makes it impossible to read the whole ITS region. In general, the sequencing of shorter amplicons tended to produce more full-length reads. However, Tonge *et al*.^19^ indicated that for NGS analysis of candidiasis, the ITS1 region rather than the ITS2 region was the preferred option. Although we also examined the oral fungal population by NGS analysis using the ITS1 region, *C. glabrata* as one of the common *Candida* species could not be identified because the length of its ITS1 region may exceed 400 bp. Moreover, some fungi detected in this study including *Exophiala equina*, *Meyerozyma guilliermondii*, and *Trichosporon cutaneum* have not previously been reported as oral fungal flora. However, *Meyerozyma guilliermondii* and *Trichosporon cutaneum* were considered to be involved in systemic mycosis, suggesting that POC might contribute to the pathogenesis of systemic mycosis^20^,^21^. NGS analysis of the ITS2 region as well as the ITS1 region may increase the sensitivity of this analysis, which may lead to establishment of an exhaustive analysis of the oral fungal population and contribute to decisions regarding the appropriate treatment for pathogenic fungi.

## Additional Information

**How to cite this article**: Imabayashi, Y. *et al*. Molecular analysis of fungal populations in patients with oral candidiasis using next-generation sequencing. *Sci. Rep.* **6**, 28110; doi: 10.1038/srep28110 (2016).

## Figures and Tables

**Table 1:** Quantification and species diversity of fungal populations.

	Control					POC		
	20′s (n = 16)	30′s (n = 21)	40′s (n = 15)	≥50′s (n = 14)	Total (n = 66)	Treatment (n = 15)		Total (n = 27)
						Before	After	
Quantification by real time PCR								
Copy number of total PCR products (log 10/μl)	5.8 ± 5.8	6.0 ± 5.9	6.2 ± 6.0	6.4 ± 6.6	6.1 ± 6.1	8.1 ± 13.4	6.1 ± 3.2^*^	7.5 ± 80^*^
Detection of *Candida* species by CHROMagar *Candida*								
Number of colonies per person	1.2 ± 4.8	2.8 ± 6.9	7.2 ± 7.9	12.1 ± 10.6	6.0 ± 8.7	36.0 ± 18.1	21.8 ± 11.4^*^	30.7 ± 31.5^*^
Total number of *Candida species*	1	1	2	2	2	5	2	3
Detection of fungal species by NGS								
Number of fungal species per person	12.2 ± 4.4	13.4 ± 3.8	13.6 ± 4.2	14.4 ± 3.6	13.3 ± 4.0	10.8 ± 9.5	14.1 ± 5.7	11.6 ± 82
Total number of fungal species	46	47	62	67	86	59	66	67

POC, pseudomembranous oral candidiasis; NGS, next-generation sequencing; FLO-G, Florid oral gel. Statistical significance of differences between patients with POC (n** = **27) and controls (n** = **66), and patients with POC before and after treatment (n** = **15) was determined by Student’s *t*-test (*P < 0.01).

**Table 2:** Detection rate of oral fungal species.

Fungal species	POC (n = 27)		Controls (n = 66)		*P*-value
*Candida albicans*	27	100.0%	66	100.0%	1.000
*Candida dubliniensis*	23	85.2%	41	62.1%	0.047*
*Candida tropicalis*	18	66.7%	51	77.3%	0.306
*Trichosporon cutaneum*	17	63.0%	32	48.5%	0.255
*Exophiala equina*	15	55.6%	55	83.3%	0.008*
*Meyerozyma guilliermondii*	12	44.4%	32	48.5%	0.820
*Ascomycota sp*	11	40.7%	17	25.8%	0.806
*Penicillium chermesinum*	11	40.7%	14	21.2%	0.072
*Candida parapsilosis*	9	33.3%	9	13.6%	0.042*
*Cladosporium halotolerans*	8	29.6%	39	59.1%	0.012*
*Malassezia restricta*	7	25.9%	31	47.0%	0.068
*Wallemia sebi*	7	25.9%	6	9.1%	0.048*
*Candida krusei*	6	22.2%	0	0.0%	0.001*
*Rhodosporidium fluviale*	6	22.2%	10	15.2%	0.545
*Rhodotorula mucilaginosa*	5	18.5%	10	15.2%	0.759
*Agaricomycetes sp*	4	14.8%	31	47.0%	0.004^*^
*Eurotiales sp*	4	14.8%	6	9.1%	0.469
*Malassezia sp*	4	14.8%	5	7.6%	0.439
*Rhodosporidium babjevae*	4	14.8%	1	1.5%	0.024*
*Alternaria alternata*	3	11.1%	5	7.6%	0.687
*Antrodiella micra*	3	11.1%	0	0.0%	0.023*
*Aspergillus penicillioides*	3	11.1%	3	4.5%	0.352
*Aspergillus sp*	3	11.1%	17	25.8%	0.166
*Basidiomycota sp*	3	11.1%	2	3.0%	0.145
*Cladosporium sphaerospermum*	3	11.1%	0	0.0%	0.023*
*Sporidiobolales sp*	3	11.1%	0	0.0%	0.023*
*Aspergillus_amstelodami*	2	7.4%	0	0.0%	0.082
*Aspergillus vitricola*	2	7.4%	0	0.0%	0.082
*Aureobasidium pullulans*	2	7.4%	2	3.0%	0.577
*Cryptococcus_diffluens*	2	7.4%	6	9.1%	1.000
*Cryptococcus_magnus*	2	7.4%	8	12.1%	0.718
*Debaryomyces_nepalensis*	2	7.4%	1	1.5%	0.201
*Fomitopsis_sp*	2	7.4%	0	0.0%	0.082
*Herpotrichiellaceae_sp*	2	7.4%	3	4.5%	0.626
*Peniophora_incarnata*	2	7.4%	0	0.0%	0.082
*Polyporaceae sp*	2	7.4%	5	7.6%	1.000

Only those with ≥5% of detection rate in patients with POC are listed.

*P < 0.05 (Fisher’s exact test and Yate’s correction).
